# Inhibition of Biofilm Formation of Foodborne *Staphylococcus aureus* by the Citrus Flavonoid Naringenin

**DOI:** 10.3390/foods10112614

**Published:** 2021-10-28

**Authors:** Qing-Hui Wen, Rui Wang, Si-Qi Zhao, Bo-Ru Chen, Xin-An Zeng

**Affiliations:** 1 School of Food Science and Engineering, South China University of Technology, Guangzhou 510641, China; 201610103987@mail.scut.edu.cn (Q.-H.W.); 201910105089@mail.scut.edu.cn (R.W.); 202120126516@mail.scut.edu.cn (S.-Q.Z.); 201811006830@mail.scut.edu.cn (B.-R.C.); 2 Overseas Expertise Introduction Center for Discipline Innovation of Food Nutrition and Human Health (111 Center), Guangzhou 510641, China; 3 School of Food Science and Engineering, Foshan University, Foshan 528000, China

**Keywords:** naringenin, biofilm formation, cell surface hydrophobicity, confocal laser scanning microscopy, biofilm-related genes

## Abstract

Taking into consideration the importance of biofilms in food deterioration and the potential risks of antiseptic compounds, antimicrobial agents that naturally occurring are a more acceptable choice for preventing biofilm formation and in attempts to improve antibacterial effects and efficacy. Citrus flavonoids possess a variety of biological activities, including antimicrobial properties. Therefore, the anti-biofilm formation properties of the citrus flavonoid naringenin on the *Staphylococcus aureus* ATCC 6538 (*S. aureus*) were investigated using subminimum inhibitory concentrations (sub-MICs) of 5~60 mg/L. The results were confirmed using laser and scanning electron microscopy techniques, which revealed that the thick coating of *S. aureus* biofilms became thinner and finally separated into individual colonies when exposed to naringenin. The decreased biofilm formation of *S. aureus* cells may be due to a decrease in cell surface hydrophobicity and exopolysaccharide production, which is involved in the adherence or maturation of biofilms. Moreover, transcriptional results show that there was a downregulation in the expression of biofilm-related genes and alternative sigma factor *sigB* induced by naringenin. This work provides insight into the anti-biofilm mechanism of naringenin in *S. aureus* and suggests the possibility of naringenin being used in the industrial food industry for the prevention of biofilm formation.

## 1. Introduction

*Staphylococcus aureus* is a common pathogen and is responsible for food poisoning through the production of thermally stable enterotoxins in various kinds of food [1,2]. A microbial biofilm is an aggregation of bacteria that is composed of extracellular polymeric substances, which are attached on the surface of microorganisms [3]. The most common feature of microbial lifestyles is attachment onto a surface by biofilm formation. Notably, *S. aureus* can form biofilms on different surfaces in food processing plants and is very adaptable to various environmental stressors including acids, salts, antibiotics, and detergents [4,5,6]. The presence of *S. aureus* biofilm on food contact surfaces creates serious problems for the food industry because it can lead to food spoilage and disease transmission [7,8]. Therefore, it is important to inhibit the formation of *S. aureus* biofilms on food contact to surfaces ensure the manufacture of safe food products.

Flavonoids from fruits and vegetables have been shown to has a range of biological activities [9,10]. For example, the citrus flavonoid naringenin have beneficial effects on human health by preventing various diseases, including diabetes, hypertension and cancer [11,12,13]. Moreover, naringenin has wide antibacterial activity and can prevent the growth of numerous microorganisms [14,15]. Specifically, naringenin from bergamot peel inhibits *Escherichia coli*, *Lactococcus lactis*, *Salmonella* Enteritidis, and *Pseudomonas putida* with minimum inhibitory concentration (MIC) values ranging from 0.25 to 1.0 g/L [9]. A small number of studies have reported that naringenin inhibits the biofilm formation of microorganisms (*Escherichia coli*, *Vibrio harveyi* and *Streptococcus mutans*) by affecting the expression of bacteria related genes and surface hydrophobicity [16,17]. In our earlier study, we determined that naringenin has strong antibacterial activity against *S. aureus* via such mechanisms of action as disrupting the bacterial cytoplasmic membrane and binding to its genomic DNA [18]. Moreover, we also found that naringenin has a strong effect in suppressing the biofilm formation of S. aureus on the surface of glass and plastic well plates. However, to the best of our knowledge, research into biofilm inhibition by naringenin is limited, and its anti-biofilm mechanism is also unclear.

Hence, we aimed to study the effect of naringenin on the inhibition of the biofilm formation of *S. aureus* at different temperatures (25 and 37 °C) using confocal laser and scanning electron microscopy techniques and exopolysaccharide production (EPS) and hydrophobicity assays. Furthermore, our study also investigated the genes (*sigB*) related to *S. aureus* biofilms using RT-qPCR, which is the main regulator of gene transcription and expression under the stress conditions induced by naringenin.

## 2. Materials and Methods

### 2.1. Bacterial Strain and Biofilm Formation

The foodborne strain *S. aureus* was obtained from the Microbial Culture Collection Center of Guangdong Institute of Microbiology (Guangzhou, China) and activated by culturing twice in 100 mL of sterile tryptic soy broth (TSB, Beijing Aoboxing Biotechnology Co., Ltd., Beijing, China) at 37 °C for 24 h. The effect of naringenin (purity ≥ 98%, Aladdin Chemical Co., Shanghai, China) on *S. aureus* growth was evaluated by transferring pre-cultured *S. aureus* cells into fresh TSB liquid medium (OD_600 nm_ ≈ 0.08) and cultivating in 96-well polystyrene plates at different temperature (25 and 37 °C) with gentle shaking. In order to measure the absorbance value of *S. aureus* growth, a FilterMax F5 multifunctional microplate reader (American molecular devices company, Sunnyvale, CA, USA) was used.

The biofilm assay was performed under similar conditions without shaking. The volume of DMSO, that was used to dissolve naringenin was equal in all of the samples, while the final concentration of naringenin varied from 0 to 60 mg/L. The crystal violet staining method was used to quantify *S. aureus* biofilm according to a relevant publication [19].

### 2.2. Cell Surface Hydrophobicity of S. aureus

The effects of naringenin on *S. aureus* cell surface hydrophobicity were evaluated at 25 and 37 °C by analyzing cells adhesion to xylene, as previously described [20]. After cultivation to the stationary-phase (48 h for *S. aureus* at 25 °C, and 12 h for *S. aureus* at 37 °C), *S. aureus* cells were collected by refrigerated centrifugation at 4000× *g* for 5 min. The *S. aureus* pellet was washed twice using distilled water and resuspended in 3 mL of a 0.85% NaCl solution (OD_600 nm_ ≈ 0.3), which defined as A_1_. Xylene (1 mL) was added to a 3 mL suspension of *S. aureus* and then incubated for 15 min at 25 °C. After vortexing for two minutes and then incubating for 15 min, the mixture separated into a xylene/water bilayer system. The OD_600 nm_ of the aqueous phase of the bilayer was recorded as A_2_. The index of cell surface hydrophobicity (I) was determined using Equation (1):

*I* = (1 − *A*_2_/*A*_1_) × 100% (1)

### 2.3. Quantification EPS Production of S. aureus

After growing in the various subminimum inhibitory concentrations of naringenin, the *S. aureus* cells were centrifuged (12,000× *g* for 15 min at 4 °C) and the supernatant of *S. aureus* was then filtered through glass fiber filters. An equal volume of absolute ethanol was then added to this supernatant of *S. aureus* and incubated overnight at 4 °C to precipitate EPS. The precipitate was resuspended in water with gentle heating (50 °C) and then quantified using a phenol–sulfuric acid procedure [21]. The percentage of EPS reduction upon exposure to naringenin was evaluated using Equation (2):

Reduction of EPS (%) = (1 − *E*_2_/*E*_1_) × 100% (2)

where *E*_1_ and *E*_2_ are the absorbances at 490 nm for *S. aureus* cells grown in the absence and presence of naringenin, respectively.

### 2.4. CLSM and SEM of S. aureus Biofilms

A 3 mL aliquot of inoculum (OD_600 nm_ ≈ 0.1) was transferred into the wells of a 6-well plate containing 13 mm-diameter sterile glass coverslips. After incubation at 25 and 37 °C for 48 h and 12 h respectively, the medium with free-floating *S. aureus* cells was removed and the coverslips washed thrice in sterile 0.85% saline solution. *S. aureus* biofilms on glass coverslips were then stained for 20 min in the dark at room temperature with diluted 5(6)-carboxy fluorescein diacetate succinimidyl ester (Aladdin Chemical Co., Shanghai, China). The stained biofilms were adjusted and photographed using a confocal laser scanning microscope (Leica, Wetzlar, Germany). At least ten pictures were taken from different locations for each sample, and the image data were then processed and analyzed.

*S. aureus* biofilms were prepared for SEM analysis as previously described, with minor modifications [18]. Glutaraldehyde (2.5% in 0.01 M phosphate buffer, pH 7.2) was added to the samples and incubated overnight at 4 °C and then dehydrated using a series of ethanol solutions (20 min each time) of increasing concentration (30~100%). The dehydrated biofilms were then incubated in tertiary butanol twice for 20 min each, followed by air-drying overnight. After gold-coating by ion sputtering (Jeol JFC-1100, Tokyo, Japan), *S. aureus* biofilms were photographed by scanning electron microscopy (SEM, Zeiss EVO18, Germany) with operation at 10.0 kV.

### 2.5. RNA Extraction and Real-Time Quantitative PCR (RT-qPCR) Analysis

TRIzol reagent (Invitrogen, CA, USA) was used according to the kit instructions to extract the *S. aureus* RNA. To check the concentration and purity, the RNA was measure at OD_260_ and OD_280_ using an 1800 UV spectrophotometer (Shimadzu Corporation, Kyoto, Japan). cDNA was reverse transcribed from 800 ng RNA with 4.0 µL of 5 × reaction buffer, 0.5 µL Thermo Scientific RiboLock RNase Inhibitor (20 U) and 1.0 µL RevertAid Premium Reverse Transcriptase (200 U), following the protocol of RevertAid Premium First Strand cDNA Synthesis Kit (Thermo Scientific™ EP0733, Thermo Fisher Scientific, Waltham, MA, USA).

RT-qPCRs was performed on an Applied Biosystems StepOne Plus™ thermocycler (Life Technologies Inc., Milano, Italy) using the SybrGreen qPCR Master Mix, following the kit instructions. Reactions were carried out in a system which was composed of 10 μL Master Mix, 0.4 μL of 0.25 μM solutions of each primer (Table 1), and 2 μL cDNA, diluted to a final volume of 20 μL using double-distilled water (DNase-free). The following thermal profile was used: a holding step for 3 min at 95 °C, followed by a cycling step consisting of 45 cycles at 95 °C for 7 s (to melt), 57 °C for 10 s (to anneal) and 72 °C for 15 s (to extend). The endogenous reference gene of 16S rRNA was used to evaluate the changes in transcriptional levels of the *S. aureus* RNA.

### 2.6. Statistical Analysis

Results are expressed as means ± standard deviation (SD), and data graphics were drawn using OriginPro 7.0 (OriginLab, Northampton, MA, USA). SPSS software (IBM, Armonk, NY, USA) was used to analyze the variance (ANOVA) by Tukey’s test, and *p* < 0.05 was represented for significant difference.

**Table 1:** Sequences of the primers used for RT-qPCR.

Gene	Primer
cidA	Forward 5′-AGCGTAATTTCGGAAGCAACATCCA-3′
	Reverse 5′-CCCTTAGCCGGCAGTATTGTTGGTC-3′
icaA	Forward 5′-CTG GCG CAG TCA ATA CTA TTT CGG GTG TCT-3′
	Reverse 5′-GAC CTC CCA ATG TTT CTG GAA CCA ACA TCC-3′
dltB	Forward 5′-GTGGACATCAGATTCACTTCC-3′
	Reverse 5′-ATAGAACCATCACGAATTTCC-3′
agrA	Forward 5′-TGATAATCCTTATGAGGTGCTT-3′
	Reverse 5′-CACTGTGACTCGTAACGAAAA-3′
sortaseA	Forward 5′-AAACCACATATCGATAATTATC-3′
	Reverse 5′-TTATTTGACTTCTGTAGCTACAA-3′
sarA	Forward 5′-CAAACAACCACAAGTTGTTAAAGC-3′
	Reverse 5′-TGTTTGCTTCAGTGATTCGTTT-3′
sigB	Forward 5′-AAGTGATTCGTAAGGACGTCT-3′
	Reverse 5′-TCGATAACTATAACCAAAGCCT-3′
16S rRNA	Forward 5′-CGGTGAATACGTTCYCGG-3′
	Reverse 5′-GGWTACCTTGTTACGACTT-3′

## 3. Results

### 3.1. Effects of Naringenin on S. aureus at Different Growth Temperatures

**Figure 1:**
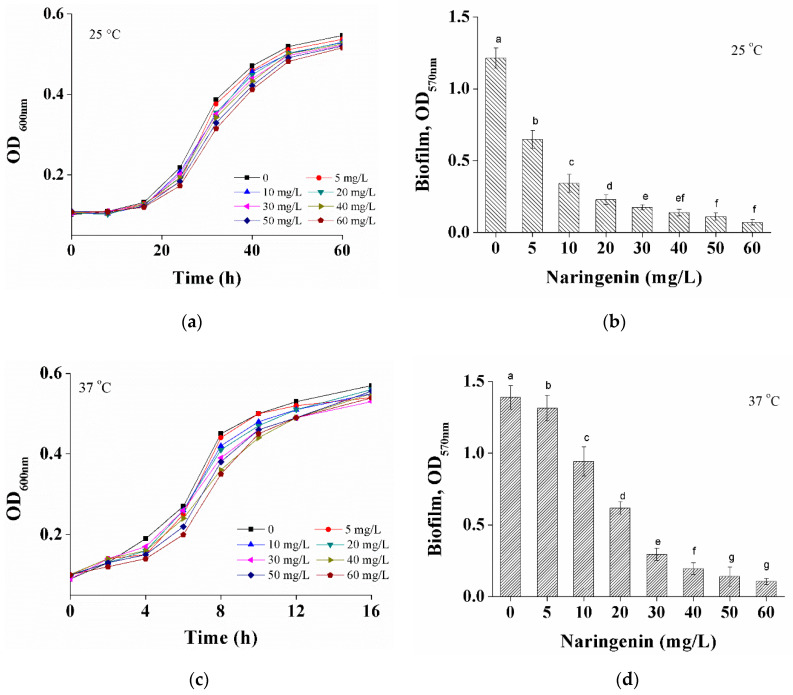
Cell growth of *S. aureus* in the presence of naringenin (0~60 mg/mL) was measured at OD_600 nm_ in 96-well plates at 25 °C (**a**) or 37 °C (**c**). Biofilm formations of *S. aureus* with naringenin concentration of 0~60 mg/mL at 25 °C (**b**) or 37 °C (**d**) for 48 and 12 h in 96-well plates, respectively. Biofilm OD values are processed as mean ± SD and a–g indicate significant differences between different columns (*p* < 0.05).

Figure 1 shows the effect of naringenin on cell growth profiles of *S. aureus* at different temperatures as reflected by the optical density (OD) at 600 nm. Subminimum inhibitory concentrations (MIC for 0.5 g/L) [22] values in the range of (5~60 mg/L) did not decrease the cell density of *S. aureus* at 25 °C (Figure 1a) and 37 °C (Figure 1c). However, the time taken to reach the stationary phase for *S. aureus* was significantly affected by temperatures. S. aureus cultured at 37 °C takes 12 h to reach the stationary phase, as compared to 48 h for *S. aureus* grown at 25 °C.

The effect of naringenin on the biofilms formed at different temperatures was measured by crystal violet staining, expressed as OD_570_ nm. Due to different growth rates, *S. aureus* at 25 and 37 °C were incubated for 48 and 12 h, respectively. *S. aureus* biofilm formation (measured at OD_570_ nm) decreased with increasing concentrations of naringenin. For example, the OD_570_ nm of S. aureus grown at 37 °C was reduced by 0.94 (32.3%) with 10 mg/mL and 0.61 (56.1%) with 20 mg/mL naringenin (*p* < 0.05). A further decrease was observed after the addition of 30 mg/mL or higher naringenin concentrations (Figure 1d). By contrast, *S. aureus* cultivated at 25 °C showed a larger decrease (*p* < 0.05) in biofilm formation (as measured by OD_570_ nm) in the presence of naringenin. For example, OD_570_ nm decreased from 1.21 to 0.22 (81.8%) after exposure to naringenin at a concentration of 20 mg/L (Figure 1b).

### 3.2. Changes in Cell Surface Hydrophobicity and EPS Production of S. aureus

**Figure 2:**
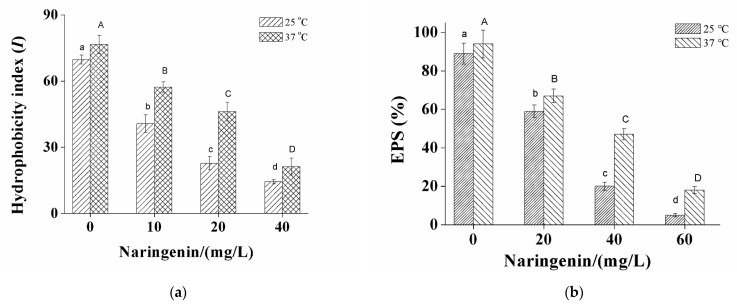
Effects of naringenin in the concentrations of 0, 20 and 40 mg/L on cell surface hydrophobicity (**a**) and EPS production (**b**) of *S. aureus*. Values are mean ± SD and there are significant differences between the values of columns marked with different letters (a–d) and (A–D), as indicated (*p* < 0.05).

Surface hydrophobicity of *S. aureus* cells was determined and expressed as hydrophobicity index (I). Figure 2a shows the significant dose-related reduction (*p* < 0.05) in cell hydrophobicity of *S. aureus* with increasing naringenin concentration at both 25 and 37 °C. Naringenin at a concentration of 10 mg/L dramatically reduced the surface hydrophobicity of *S. aureus* cells grown at 25 and 37 °C by 40.6% and 57.2% (*p* < 0.05), respectively. The respective values further decreased to 14.4% and 21.2% (*p* < 0.05) when the concentration of naringenin was increased to 40 mg/L.

The effect of naringenin on the EPS production of *S. aureus* was also investigated. The results reveal that *S. aureus* treated with various concentrations of naringenin (20, 40, and 60 mg/L) show a significant reduction (*p* < 0.05) in EPS compared to that of the control (Figure 2b). For *S. aureus* grown at 25°C, naringenin at 20 and 60 mg/mL reduced EPS by 59% and 5%, respectively. At 37 °C, the same concentrations of naringenin reduced EPS by 67% and 18%, respectively.

### 3.3. Microscopic Observations of S. aureus Biofilm

**Figure 3:**
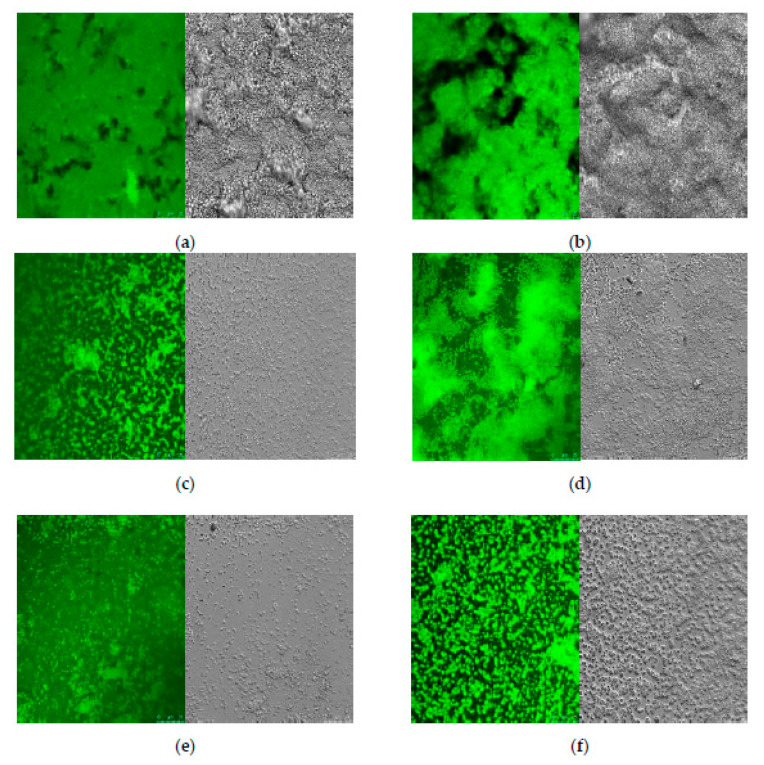
Confocal laser scanning microscopy (CLSM) analysis of biofilms formed by *S. aureus* incubated with different concentrations of naringenin. (**a**,**c**,**e**) for *S. aureus* cells were grown at 25 °C with naringenin at 0, 20 and 40 mg/L, respectively; (**b**,**d**,**f**) for *S. aureus* cells were grown at 37 °C with naringenin at 0, 20 and 40 mg/L, respectively.

Direct visual information, including the surface coverage and thickness of *S. aureus* biofilms, were obtained by CLSM analyses. As shown in Figure 3a,b, *S. aureus* formed thick and compact biofilms covering the surface of glass coverslips at 25 and 37 °C when grown in the absence of naringenin. The confocal stack images show that the thick coating of *S. aureus* biofilms represented by cell aggregations became thinner and looser on the surfaces in the presence of 20 mg/L naringenin (Figure 3d). At 40 mg/L of naringenin, there was a visible reduction in the numbers of microcolonies for *S. aureus* cells grown at 37 °C (Figure 3f). Compared to 37 °C, the cells grown at 25 °C had a more obvious decrease associated with naringenin exposure, and the bacterial density was significantly decreased (Figure 3c,e). These results were further confirmed by SEM images.

**Figure 4:**
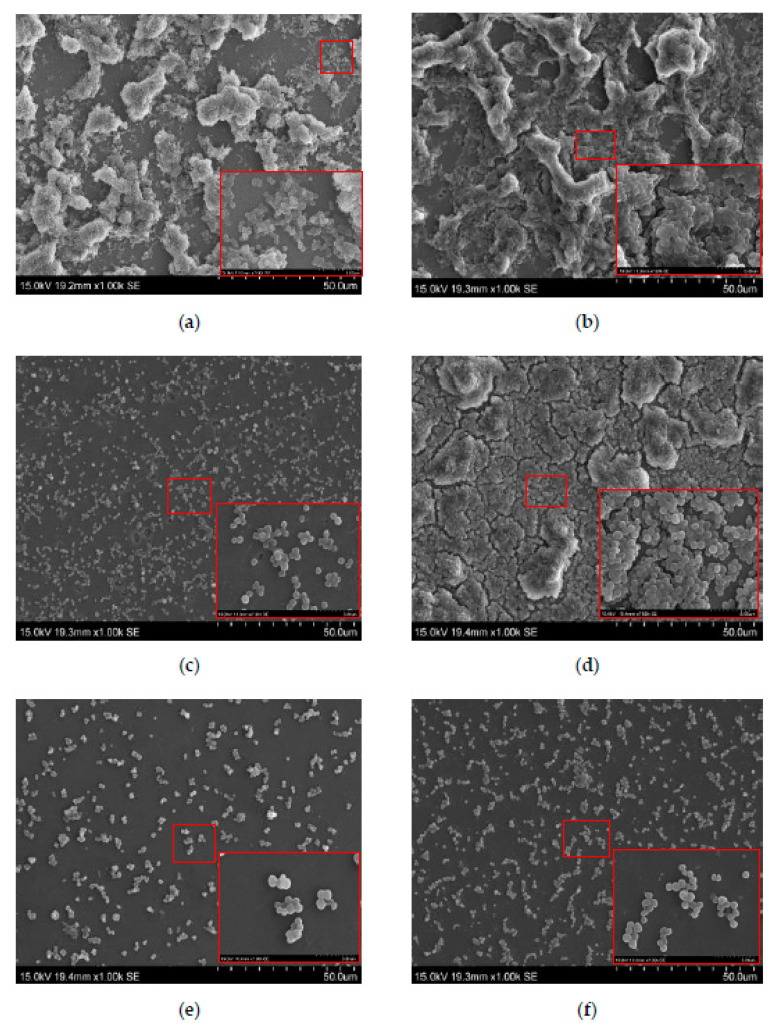
SEM images showing inhibitory activity of naringenin on biofilm formation of *S. aureus* cells. (**a**,**c**,**e**) for *S. aureus* cells grown at 25 °C with naringenin at 0, 20 and 40 mg/L, respectively; (**b**,**d**,**f**) for *S. aureus* cells grown at 37 °C with naringenin at 0, 20 and 40 mg/L, respectively.

The SEM images show that naringenin inhibited the bacterial growth of *S. aureus* at subminimum inhibitory concentrations (sub-MICs) values of 0, 20 and 40 mg/L. As the concentration of naringenin increases, the total number of bacteria obviously decreased, especially at high concentrations (Figure 4e,f) [17]. Compared to the incubation temperature of 37 °C (Figure 4b,d,f), the total number of *S. aureus* cultivated at 25 °C (Figure 4a,c,e) showed a greater decrease, demonstrating that naringenin has a significant effect in suppressing the biofilm formation of bacteria.

### 3.4. Transcriptional Analysis of Biofilm-Related Genes in S. aureus Cells

**Figure 5:**
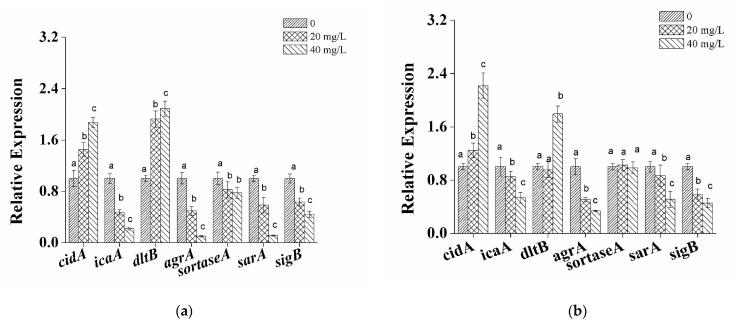
Effect of naringenin on the expression levels of biofilm-related genes in *S. aureus* at 25 °C (**a**) and 37 °C (**b**), where 16S rRNA was used as a reference gene. Data are presented as means ± standard deviations. Relative expression values are processed as mean ± SD and a–c indicate significant differences between different columns (*p* < 0.05).

The effect of naringenin on the level of expression of biofilm-related genes, including *cidA*, *icaA*, *dltB*, *agrA*, *sortaseA*, *sarA* and sigma factor *sigB* in *S. aureus* cells, were studied by RT-qPCR. Among the seven tested genes, *icaA*, *agrA*, *sarA* and *sigB* demonstrated significantly down-regulated (*p* < 0.05) gene expression when treated with naringenin, while *cidA* and *dltB* were up-regulated. Specifically, *icaA*, *agrA*, *sarA* and *sigB* were significantly down-regulated (*p* < 0.05) by 0.47-, 0.49, 0.58- and 0.63-fold for *S. aureus* grown at 25 °C in a culture medium with 20 mg/L naringenin (Figure 5a), and further decreased by 0.22-, 0.10-, 0.11- and 0.44-fold after the concentration of naringenin was increased to 40 mg/L, respectively. Under the same conditions, the expression of *cidA* and *dltB* were mildly up-regulated by 1.29- and 2.08-fold when *S. aureus* cells were exposed to naringenin at 40 mg/L. The expression of genes, including *cidA*, *dltB*, *icaA*, *agrA*, *sarA* and *sigB*, exhibited a similar trend for *S. aureus* cells cultivated at 37 °C (Figure 5b).

By contrast, the levels of *sortaseA* expression in *S. aureus* cells grown at 25 °C were down-regulated in the presence of naringenin at concentrations of 20 and 40 mg/L, exhibiting 0.82- and 0.78-fold decreases (*p* < 0.05), respectively. At 37 °C, naringenin had no obvious effect on the transcription level of the studied *sortaseA* genes.

## 4. Discussion

In this work, no significant inhibitory effect of naringenin on S. aureus biofilm formation at 25 and 37 °C was found. However, the growth rate of S. aureus at 25 °C was significantly lower than that at 37 °C, without the final biomass being affected. Thus, different incubation times, i.e., 48 and 12 h, were used for 25 and 37 °C, respectively, to compensate for variations in the time required to reach the stationary phase. At subminimum inhibitory concentrations (sub-MICs) values ranging from 5 to 60 mg/L, naringenin dramatically inhibited *S. aureus* biofilm formation, with the biofilm formation further decreasing as the concentrations of naringenin with increased. Compared to an incubation temperature of 37 °C, the presence of naringenin resulted in a more significant effect on *S. aureus* biofilm formation for cultivation at 25 °C. There are various incubation temperatures that affect the growth of *S. aureus* and a series of changes caused by differences in subsequent growth.

Cell surface hydrophobicity is an important physical-chemical property of bacteria that facilitates their attachment to surfaces. Previously, it was reported that bacterial adherence of oral streptococci to the tooth surface was significantly suppressed by the reduction in cell surface hydrophobicity after treatment with constituents in cranberry juice and tea extract polyphenols [23,24]. Our results indicated that the cell surface hydrophobicity of *S. aureus* is reduced by treatment with naringenin (Figure 2a).

EPS is also important for biofilm production, forming multiple layers that help pathogens to adhere to surfaces and maintain biofilm architecture and acting as a protective barrier to prevent the entry of antibacterial agents into bacterial cells [25,26]. Therefore, the substantial decrease in EPS production by *S. aureus* after treatment with naringenin (Figure 2b) is consistent with this scenario. Since EPS production and surface hydrophobicity are important factors for biofilm formation, the decreases in these two properties following treatment with naringenin are the likely reasons that naringenin decreases biofilm formation in *S. aureus* cells.

Naringenin is not an antibiotic and, thus, the results obtained from CLSM and SEM are inconsistent with some previous studies that have reported that low concentrations of β-lactam and aminoglycoside antibiotics often facilitate biofilm formation by bacteria [27,28]. However, the inhibition of biofilm formation by naringenin is in agreement with the behavior of other flavonoids, including morin, rutin, quercetin and phloretin [25,29,30,31]. Notably, naringenin has a better effect on inhibiting the formation biofilms of *S. aureus* at the incubation temperature at 25 than at 37 °C.

To elucidate the underlying molecular mechanism for the inhibition of *S. aureus* biofilm by naringenin, we investigated the expression of some biofilm-related genes using RT-qPCR, including *dltB*, *sarA*, *sortaseA*, *agrA*, *icaA*, *cidA* and *sigB*. The *dltB* gene is responsible for D-alanylation of teichoic acids and the translocation of D-alanine through the cell membrane. It has been previously reported that *dltB* deficiency results in a higher negative net charge on the bacterial cell surface and defects in the initial binding of bacteria to a surface in the process of biofilm formation [32]. The expression of *cidA* was reported to be associated with extracellular DNA release, which is essential in the formation of *S. aureus* biofilm [33]. Our results revealed that the *dltB* and *cidA* genes are mildly up-regulated in *S. aureus* cells exposed to naringenin at 25 and 37 °C. Thus, it can be inferred that the suppression of biofilm formation by naringenin is probably not through *dltB* and *cidA* in *S. aureus*.

By contrast, the *icaA*, *agrA* and *sarA* genes are down-regulated in the presence of naringenin. The *ica* operons encode enzymes involved in the biosynthesis of polysaccharide intercellular adhesion [34]. Since it is well-known that the ability of *S. aureus* to form biofilm is dependent on polysaccharide intercellular adhesion, down-regulation of *icaA* likely decreases the production of polysaccharide intercellular adhesion, leading to reduction of biofilm formation. Thus, the decreased expression of *icaA* by naringenin might lead to a reduction in the biosynthesis of polysaccharide intercellular adhesion, which hinders the attachment of *S. aureus* cells to solid surfaces and subsequent biofilm formation. Both the *agr* (accessory gene regulator) and *sar* (staphylococcal accessory regulator) operons are two regulatory elements that control the production of virulence factors, as well mediate *S. aureus* biofilm formation by regulating the quorum sensing and polysaccharide intercellular adhesion production [35]. Thus, it can also be inferred that the down-regulated gene expression of *agrA* and *sarA* negatively affects biofilm formation by *S. aureus*. These data are in agreement with previous studies showing that *agr* down regulation has a negative impact on biofilm development by *S. aureus* [33,36].

Additionally, the expression levels of *sigB* were reduced for *S. aureus* cells exposed to naringenin. *sigB* is an alternative sigma factor modulating various stress responses in several Gram-positive bacteria, including *S. aureus* via a large regulon [37]. Overexpression of these genes can confer more resistance to heat, oxidative and antibiotic stresses. In summary, down-regulating the above genes may be the mechanism of action by which naringenin inhibits biofilm formation.

In some Gram-positive bacteria, including staphylococci, enterococci and streptococci, the *sortaseA* gene is responsible for coding a membrane enzyme that plays an important role in grappling some surface-exposed proteins to the cell wall envelope [38]. Recent reports have shown that *sortaseA* upregulation significantly increases the levels of biofilm formation in staphylococcal strains [39]. Our results show a mild down-regulation of *sortaseA* in the presence of naringenin for *S. aureus* grown at 25 °C. By contrast, at 37 °C no obvious change in the transcription level of *sortaseA* was observed. Since adhesion to the surface is an essential step for biofilm formation, the larger decrease in biofilm formation observed in *S. aureus* cultivated at 25 °C may be attributed to the down-regulated expression of *sortaseA* in *S. aureus* cells exposed to naringenin.

## 5. Conclusions

The data from our investigation indicates the potential of naringenin as a natural agent to prevent biofilm formation of S. aureus and possibly reduce health risks related to biofilm-formation in the food industry. However, more studies are necessary to gain a better understanding whether of there is any anti-biofilm activity toward other food-borne pathogens in food industry, and the efficiency when considering stainless steel and plastic surface.

## Data Availability

Not applicable.
